# Invasive Mold Infection of the Central Nervous System in Immunocompromised Children

**DOI:** 10.3390/jof6040226

**Published:** 2020-10-16

**Authors:** Luciana Porto, Se-Jong You, Andishe Attarbaschi, Gunnar Cario, Michaela Döring, Olga Moser, Urs Mücke, Fiona Poyer, Christian Temme, Sebastian Voigt, Andreas H. Groll, Melchior Lauten, Elke Hattingen, Thomas Lehrnbecher

**Affiliations:** 1 Department of Neuroradiology, University of Frankfurt, 60528 Frankfurt, Germany; se-jong.you@kgu.de (S.-J.Y.); elke.hattingen@kgu.de (E.H.); 2 Department of Pediatrics and Adolescent Medicine, St. Anna Children’s Hospital, Medical University of Vienna, Pediatric Hematology and Oncology, 1090 Vienna, Austria; andishe.attarbaschi@stanna.at (A.A.); fiona.poyer@stanna.at (F.P.); 3 Department of Paediatrics, Pediatric Hematology and Oncology, Christian Albrechts University Kiel, 24118 Kiel, Germany; gunnar.cario@uksh.de; 4 Department of Paediatric Haematology and Oncology, University Children’s Hospital Tübingen, 53424 Tübingen, Germany; michaela.doering@med.uni-tuebingen.de; 5 Department of Paediatrics, University Hospital Aachen, 52056 Aachen, Germany; omoser@ukaachen.de; 6 Department of Paediatric Haematology and Oncology, Hannover Medical School, 30625 Hannover, Germany; muecke.urs@mh-hannover.de; 7 Department of Paediatric Haematology and Oncology, University Hospital Essen, 45147 Essen, Germany; christian.temme@uk-essen.de; 8 Department of Paediatric Haematology and Oncology, University Hospital Charité Berlin, 10117 Berlin, Germany; sebastian.voigt@charite.de; 9 Department of Infectious Disease Research, Center for Bone Marrow Transplantation and Department of Pediatric Hematology/Oncology, University Children’s Hospital Münster, 53424 Münster, Germany; grollan@ukmuenster.de; 10 Department of Paediatrics, Paediatric Haematology and Oncology, University of Lübeck, 23562 Lübeck, Germany; melchior.lauten@uksh.de; 11 Department of Pediatric Hematology and Oncology, Hospital for Children and Adolescents, University of Frankfurt, 60528 Frankfurt, Germany; thomas.lehrnbecher@kgu.de

**Keywords:** child, invasive mold infection, central nervous system, magnetic resonance imaging of the brain, outcome

## Abstract

*Background:* Due to the difficulties in the definite diagnosis, data on brain imaging in pediatric patients with central nervous system (CNS)-invasive mold infection (IMD) are scarce. Our aim was to describe brain imaging abnormalities seen in immunocompromised children with CNS-IMD, and to analyze retrospectively whether specific imaging findings and sequences have a prognostic value. *Methods:* In a retrospective study of 19 pediatric patients with proven or probable CNS-IMD, magnetic resonance imaging (MRI)-findings were described and analyzed. The results were correlated with outcome, namely death, severe sequelae, or no neurological sequelae. *Results*: 11 children and 8 adolescents (11/8 with proven/probable CNS-IMD) were included. Seven of the patients died and 12/19 children survived (63%): seven without major neurological sequelae and five with major neurological sequelae. Multifocal ring enhancement and diffusion restriction were the most common brain MRI changes. Diffusion restriction was mostly seen at the core of the lesion. No patient with disease limited to one lobe died. Perivascular microbleeding seen on susceptibility weighted imaging (SWI) and/or gradient-echo/T2* images, as well as infarction, were associated with poor prognosis. *Conclusions:* The presence of infarction was related to poor outcome. As early microbleeding seems to be associated with poor prognosis, we suggest including SWI in routine diagnostic evaluation of immunocompromised children with suspected CNS-IMD.

## 1. Introduction

Despite the availability of potent antifungal compounds, invasive fungal infections, in particular those affecting the central nervous system (CNS), are associated with high morbidity and mortality in immunocompromised patients. Filamentous fungi, mostly *Aspergillus* spp. but also *Fusarium* spp. or mucormycetes, may affect the CNS of both children and adults. These infections can originate from hematogenous dissemination from a distant primary focus, commonly the lung, or from local dissemination, usually from a sinus infection. Clinical symptoms are often uncharacteristic and may even be absent in a significant proportion of patients with invasive mold disease (IMD) of the CNS, which makes early diagnosis difficult [1]. On the other hand, early diagnosis and treatment of IMD of the CNS seems to be associated with better outcomes. A definite diagnosis of CNS-IMD requires a microscopic finding or a positive culture from a CNS specimen, which, however, often remain negative [2]. Non-culture based diagnostic assays such as the detection of the cell wall component galactomannan by ELISA or of fungal nucleic acids by PCR in CNS specimens may support the diagnosis of CNS-IMD but require invasive procedures. Therefore, non-invasive procedures such as brain imaging studies play a key role in the early detection of CNS-IMD. Unfortunately, due to the difficulties in the definite diagnosis of CNS-IMD, primary data on imaging studies such as cranial magnetic resonance tomography (MRI) in proven and probable CNS-IMD in the pediatric age group are scarce, reporting only on single patients [3] or small case series [4] or on patients with only clinical features of CNS-IMD [5], which is clearly a major limitation of these analyses. Data from studies in adult patients may not be applied to children, as there are important differences between children and adults regarding underlying malignancy, comorbidities, treatment strategies and immunologic recovery, all of which might impact the results of imaging studies [6]. We therefore aimed to describe the brain imaging abnormalities seen in immunocompromised children with CNS-IMD, and to analyze whether specific MRI findings and sequences have a prognostic value.

## 2. Materials and Methods 

For this retrospective study, children and adolescents diagnosed between 2007 and 2016 with CNS-IMD were identified by the recollection of local investigators. The detailed analyses of diagnostic procedures, therapy, clinical course, and outcome (e.g., death or severe neurological sequelae such as hemi-/quadriplegia, aphasia, amaurosis) have been reported previously [1,7]. For the present analysis, pediatric patients receiving chemotherapy for an underlying malignancy and patients undergoing allogeneic hematopoietic stem cell transplantation (HSCT) were included if they met the following criteria: (1) younger than 18 years at the time of diagnosis; (2) proven or probable CNS-IMD, as defined previously [1]; and (3) availability of (a) non-contrast T1-weighted images (T1-W), (b) T2-W, (c) fluid-attenuated inversion recovery sequences (FLAIR), (d) gradient-echo T2* and/or susceptibility weighted imaging (SWI), (e) diffusion-weighted imaging (DWI) with apparent diffusion coefficient (ADC) maps, and (f) contrast-enhanced T1-W in at least two different planes and without severe motion artefacts. 

Proven CNS-IMD was diagnosed by compatible CNS imaging or macroscopic autopsy findings in conjunction with a positive microbiological result in the brain tissue or cerebrospinal fluid [1]. Positive microbiological results included a positive culture, microscopic evidence of hyphae, a positive result of the galactomannan assay (OD > 0.5) or detection of a mold by polymerase chain reaction (PCR). Probable CNS-IMD was defined as compatible CNS imaging findings in combination with proven or probable IMD at a site outside the CNS in the absence of a plausible alternative diagnosis [1]. Patients with possible CNS-IMD (e.g., patients without microbiological result of IFD in or outside the CNS) were not included into the analysis. 

The MRI sequences were analyzed as follows:

(1) DWI and ADC were evaluated for infarction, lesions with central restricted diffusion, lesions with a rim of restricted diffusion, and others. Infarction was defined as territorial, cortical, and sub-cortical lesions without nodular or ring-like aspect. Non-infarction-DWI restrictions, with nodular or ring-like aspect, were classified as lesions with central or rim-like restricted diffusion. Others included patients with cerebritis and bleeding.
(2) SWI/T2* were evaluated as follows:
  - Parenchymal bleeding was defined as a hypointense parenchymal signal with “blooming”.
  - Cluster of microbleeding was defined as a significantly hypointense cluster signal.
  - Perivascular microbleeding was defined as a significantly hypointense perivascular signal.
  - Ring-like was defined as a hypointense ring-like signal.
  - Vascular blooming was defined as a vascular structure with signal loss and prominent “blooming” on SWI/T2*w sequences.
(3) Focal or multifocal involvement as well as the type of enhancement on T1 images after contrast were evaluated.

Due to the multicenter approach, MRI studies were performed with MR scanners of different manufacturers at 1.5- to 3.0-Tesla field strengths. Two senior neuroradiologists (L.P. and S.Y.) described and analyzed the radiological findings of the lesions according to consensus. 

The study was reviewed and approved by the Ethics Committee of the University of Lübeck (vote no. 15-301, 05.11.2015).

## 3. Results

### 3.1. Patient Demographics and Outcome

A total of 19 out of 29 children and adolescents included in the original analysis had sufficient MRI imaging sequences and met the criteria for this study. The 5 girls and 14 boys were between 2 and 18 years (median age, 12 years) (Table 1). The patients suffered from de novo or relapsed acute lymphoblastic leukemia (ALL, *n* = 12 and *n* = 2, respectively), myelodysplastic syndrome (MDS), T-non-Hodgkin lymphoma (T-NHL), chronic myelogenous leukemia (CML), severe combined immunodeficiency (SCID), and chronic granulomatous disease (CGD) (one patient each). Of the 19 children, 7 died (37%), 7 (37%) showed no neurological sequelae, and 5 (26%) severe neurological sequelae in the follow-up. The deceased patients suffered from ALL (*n* = 4), SCID, CGD, and MDS (*n* = 1 each) and were between 3 and 18 years old. In all of them, the fungal infection was the cause of death. All 5 children with severe neurological sequelae had ALL as a primary disease; age varied between 3 and 14 years.

*Aspergillus* spp. was found in 10 patients (one patient had a co-infection with *Rhizopus arrhizus*) and one patient suffered from *Rhizomucor pusillus*. In five patients, hyphae were detected in the CNS sample, and three patients met the criteria for probable CNS-IMD without an isolated pathogen. Central nervous system IMD was proven in 11 and probable in 8 patients (Table 1). In all but one patient, proven or probable IMD was also diagnosed outside the CNS. 

Of the seven patients who died, *A. fumigatus* and *Rhizomucor pusillus* were isolated in three and one patients, respectively, and hyphae were detected in two patients. Of the five patients with severe neurological sequela, *A. fumigatus* and hyphae were detected in two each (one patient suffered from co-infection with *A. fumigatus/Rhizopus arrhizus*). *A. fumigatus* was detected in five of the seven patients who survived without severe neurological sequelae, and in one patient, hyphae were detected.

### 3.2. Diffusion Imaging Aspects and Outcome

Restriction in diffusion was always confirmed on ADC maps. DWI and ADC demonstrated infarction in seven patients (Table 1 and Table 2). Three of the seven patients with infarction died, and three others had severe neurological sequelae (those who deceased were between three and seven years of age, and those with severe residual sequelae were between 5 and 14 years of age). Only one patient (17 years) had a favorable outcome, with the infarction restricted to a small area due to a focal pericallosal vasculitis. Out of the 7 patients with infarction, one patient suffered from *A. fumigatus* and *Rhizopus arrhizus* co-infection, one from *Rhizomucor pusillus* infection, and in the remaining five patients, hyphae have been detected in the CNS. 

The most common type of non-infarction-DWI restrictions was a central pattern (*n* = 10), i.e., at the core of the lesion. Rim restricted diffusion was seen in three patients. There was no correlation between the pattern of restricted diffusion and outcome (Table 2).

### 3.3. T2*, SWI Aspects Compared to Outcome 

T2*, SWI findings demonstrated bleeding in 12 of the 19 pediatric patients with CNS-IMD (Table 3). The MRI aspect in these patients varied from cerebral microbleeds to cluster or perivascular microbleeding, or parenchymal bleeding. The most frequent MRI finding on T2* W images and SWI was a hypointense rim (*n* = 9), which was evenly distributed among the different groups and was not associated with poor prognosis (four of these patients died, two had severe and three no neurological sequelae, respectively).

Perivascular microbleeding was seen in 3 out of 19 patients, but when present, was related to poor outcome (two patients died and one had severe neurological sequelae). In these three patients, *A. fumigatus*/*Rhizopus arrhizus* and *Rhizomucor pusillus* were isolated, whereas in one patient, hyphae were detected which could not be further specified.

### 3.4. Parenchymal Abnormalities: Focal and Multifocal Involvement and Outcome

The analysis of sites of involvement and of enhancement in T1 images after contrast demonstrated that multifocal involvement was not related to poor outcome (Table 4). Of the 15 children with multifocal involvement, 7 died and 2 suffered from severe neurological sequelae, whereas 6 had a favorable outcome. Neither of the two patients with a single lobe involvement died. 

Multifocal ring enhancement was the most common enhancement pattern seen in immunocompromised children with CNS-IMD (*n* = 14) and was evenly distributed among children who died (*n* = 5) and those with severe (*n* = 3) or no (*n* = 6) neurological sequelae. Nodular enhancement was not related with poor prognosis, demonstrated in one child in each of the different subgroups (death, severe neurological sequelae, and no neurological sequelae, respectively). Subependymal enhancement was seen in two patients, and both had a poor outcome (one died, one had severe neurological sequelae). In both children, hyphae were detected in the CNS, but no pathogen could be specified.

## 4. Discussion

Central nervous system involvement of IMD is seen especially in immunocompromised patients and is associated with severe morbidity and high mortality [1,2]. As initial symptoms may be uncharacteristic and subtle, CNS-IMD is often not diagnosed at an early time point. Unfortunately, despite improved diagnostic tools, definitive diagnosis remains difficult, which also explains the fact that the exact incidence of CNS-IMD is unknown [2]. As up to one third of patients with pulmonary IMD are neurologically asymptomatic despite CNS involvement [1], recent pediatric specific guidelines recommend routine CNS imaging in patients with proven or probable mold infection outside the CNS [8]. 

### 4.1. Interpreting MRI Imaging of Invasive Mold Infection in Children—Diffusion Imaging

DWI is valuable not only in early diagnosis of CNS-IMD as it detects early infarction, but can also be beneficial in differentiating these lesions from other entities, such as neoplasms. The presence of signs of infarction demonstrated in DWI and ADC may indicate a poor outcome, since out of the seven patients with infarction, only one survived without severe neurological sequelae. 

**Figure 1:**
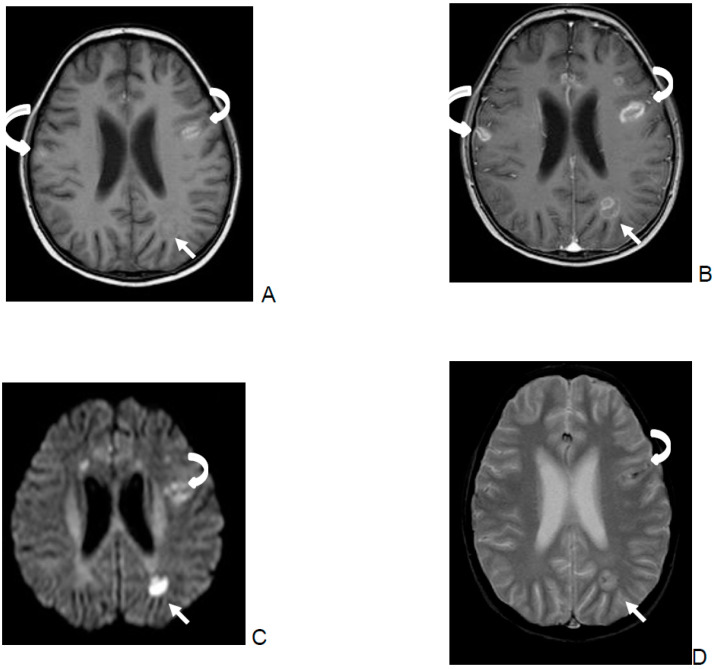
Multiple foci of hemorrhage with cerebritis and abscess formation in a 10-year-old boy with acute lymphoblastic leukemia. The patient died due to the fungal infection. The figure demonstrates the difficulties in differentiating between abscesses and parenchymal bleeding (curved arrows). 1. T1-W without contrast (**A**) reveals multiple hyperintense foci of hemorrhage (curved arrows) with gadolinium enhancement of magnetic resonance imaging (MRI) lesions on T1-W image after contrast (**B**). Enhancement in areas of parenchymal bleeding may have different patterns, such as ring, focal, and diffuse–patchy. Because of the hemorrhagic nature of the lesions, a hypointense rim or foci are usually present in SWI or GRE T2* (**D**). Factors such as oxyhemoglobin in the hyperacute stage or extracellular methemoglobin in the late sub-acute stage affect how the DWI (**C**) signal appears in the presence of hemorrhage. These substances are thought to give parenchymal hemorrhage the hyperintense appearance on DWI (curved arrow, **C**) and low ADC values, which is similar, but usually not as strong as the restricted diffusion seen in an abscess (straight arrow, **C**). 2. The abscess (white straight arrow) shows a small hyperintensity in T1-W without contrast (A), clear restricted diffusion within the core of the lesion (straight arrow, **C**) with a ring like enhancing lesion (**B**) and hypointense foci in GRE T2* (**D**). Brain abscesses due to a fungus are usually hypointense on the T1-W image before contrast, but show small areas of hyperintensity due to the presence of iron, manganese, or methemoglobin (**A**).

In children with CNS-IMD, the most common type of non-infarction-DWI restrictions was the central pattern, i.e., at the core of the lesion (Figure 1C). However, there was no correlation between the pattern of restricted diffusion and outcome.

**Figure 2:**
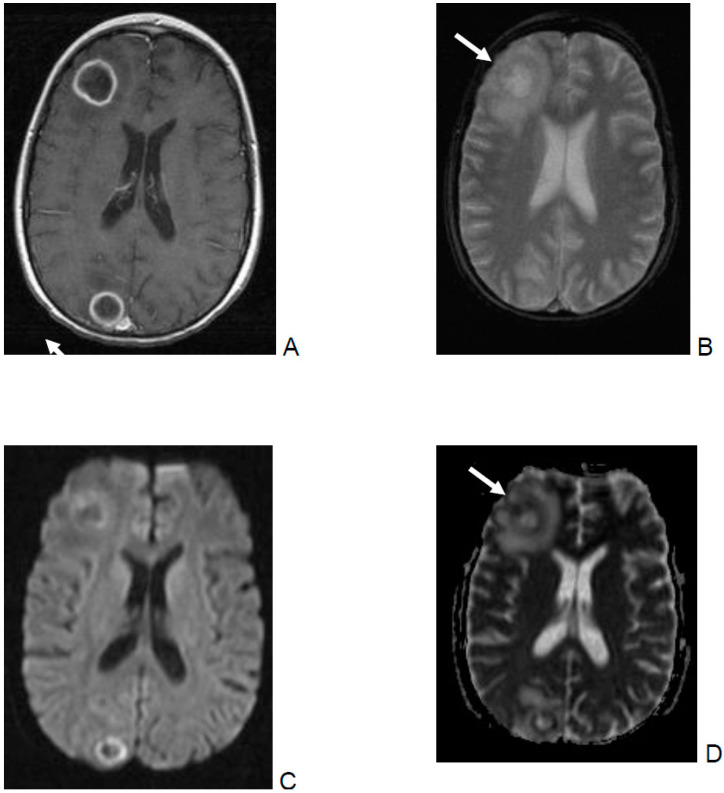
Multiple abscesses in the right hemisphere of a 15-year-old boy with non-Hodgkin lymphoma. Culture grew *Aspergillus fumigatus*. The patient is alive with no neurological sequelae. Two lesions are seen with a slightly hypointense wall on axial T2* image (**B**), which show well-defined ring enhancement on the post-contrast T1-W image (**A**). Diffusion, b = 1000 s/mm^2^ (**C**) shows ring-like hyperintensity with low ADC (0.54 × 10−3 mm^2^/s) (**D**). Note that fungal abscesses may have a variable imaging appearance but are often multiple and near the grey–white matter junction. The pattern of diffusion restriction of the wall without central restriction correlates more with a fungal abscess than a pyogenic abscess. On DWI/ADC (**C** and **D**) the walls as well as the small nodular soft tissue (straight arrows) display diffusion restriction, while the contents do not show restriction. There is perilesional edema.

Unfortunately, bleeding within the lesions complicates the interpretation of diffusion images (Figure 1D). In the present analysis, fungal abscess was defined as a nodular (DWI restriction at the core of the lesion, Figure 1C) or ring-like lesion with rim restricted diffusion restriction (Figure 2C,D). In immunocompromised patients, fungal abscesses are often multiple and near the grey–white matter junction due to the hematologic spread of the pathogen (Figure 2) and are usually hypointense on the T1-W image [9]. Due to the presence of iron, manganese, or methemoglobin, areas of high signal intensity on unenhanced T1-W images may be seen (Figure 1A). This fact often makes the differential diagnosis with superimposed hemorrhage difficult (Figure 1A,D).

Although imaging studies may help to differentiate between fungal and pyogenic abscesses, imaging findings are unfortunately non-specific for a certain pathogen [10,11]. However, the pattern of diffusion restriction of the wall without central restriction correlates with a fungal abscess rather than with a pyogenic abscess (Figure 2C,D). In this respect, the nodular soft tissue seen along the medial walls of cystic lesions is believed to consist of fungal hyphae, and the low T2 signal of the lesion walls is supposed to be due to increased iron in fungal elements [9,11].

Pathologically, fungal brain abscesses often show diffuse infiltration of the brain parenchyma with fungal hyphae beyond the fibrous capsule, and therefore the adjacent brain parenchyma demonstrates hyperemic changes with granulomas containing fungi, necrotic debris, acute and chronic inflammatory cells, as well as multinuclear giant cells [12]. This may explain the reported restricted diffusion at the wall and within the wall projections and the usual absence of restricted diffusion within the core of the lesion [13]. 

### 4.2. Interpreting MRI Imaging of Invasive Mold Infection in Children—T2*/ SWI Aspects

The SWI/T2* findings (Figure 1D) indicated parenchymal or microbleeding in 9 of the 19 children, which is not surprising, as *Aspergillus* spp. is angio-invasive. The lesions are located not only in the grey–white matter zone, but, due to the occlusion of smaller perforating arteries, are also found in the basal nuclei, thalami, corpus callosum, and brainstem. Previous reports describe hemorrhagic changes in 13% to 39% of patients with cerebral aspergillosis [14,15,16]. However, these studies did not include SWI images, which is a more sensitive sequence for bleeding. Consequently, the incidence of microbleeds is most likely underestimated in these reports. Nine of the analyzed children showed lesions with a clear or discrete rim-like hypointense signal in the T2* and/or SWI (Figure 2B), but it is important to note that differentiation between a hypointense signal around an abscess or bleeding is difficult. The former is probably associated with the presence of ferromagnetic fungal deposits, methemoglobin in the capsule wall, or free radicals produced by macrophages [15].

In our patient cohort, the presence of parenchymal bleeding was not associated with a poor outcome. In contrast, two of the three patients with perivascular microbleeding died, and one had severe neurological sequelae (left-sided hemiparesis). Therefore, it is important to recognize microbleeding, i.e., cluster and perivascular microbleeding in SWI at an early time point. Microbleeding can be the first sign of a vasculitis, or it may precede major bleeding complications. Besides bleeding, focal punctiform hypointensities on SWI/T2* may be explained by the presence of iron, manganese, and magnesium within the fungal infiltration. This could prove to be a good warning sign for vascular involvement as a complication of fungal infiltration.

Although SWI images are time consuming, our data suggest that including these sequences in the routine evaluation of children with suspected CNS-IMD may help in the early diagnosis of CNS-IMD and may be the first warning sign for later complications such as infarction or major bleeding.

### 4.3. Interpreting MRI Imaging of Invasive Mold Infection in Children—Parenchymal Involvement and Outcome 

Parenchymal involvement of the infection was multifocal in most of the patients and was limited to one lobe in only two patients. Both of these patients survived. Fungal parenchymal disease can present as granuloma, cerebritis, and/or abscess formation [2]. None of the analyzed patients showed granuloma formation, which is uncommon and nonspecific. Cerebritis usually shows as hyperintensity in T2-W and FLAIR with variable enhancement in children (no, minimal, or clear enhancement), but it is often difficult to distinguish cerebritis from perilesional edema. In our patient population, T2-hyperintensity without ring-like lesion was seen in only three patients and was related to cerebritis without abscess, infarction, and hypoxia, respectively (one patient each).

The kind of enhancement, e.g., ring or nodular enhancement, was not related with poor prognosis. Ring-like enhancement (Figure 2A), which was observed in 14 of the 19 analyzed patients, was always associated with perilesional FLAIR-hyperintensity, which may be interpreted as edema with probable infiltration of the adjacent brain parenchyma.

Subependymal enhancement without ventriculitis was seen in two patients, and can be associated with poor outcomes as one of these patients died and the other had severe neurological sequelae.

We acknowledge the fact that our study has some limitations, such as the retrospective nature of the analysis and the fact that not all children of the study period were systematically enrolled, which is a potential bias. It also remains unclear whether a biopsy ultimately altered the clinical course of a patient, and either ameliorated or deteriorated the outcome. Although the absolute number of analyzed patients seems to be relatively small, our study includes one of the largest datasets of pediatric patients suffering from proven and probable CNS-IMD reported to date, which is a strength of the analysis. In addition, the analysis of MRI imaging were performed by two senior neuroradiologists, which provides more reliable results.

## 5. Conclusions

Our retrospective analysis of 19 immunocompromised children with CNS-IMD demonstrates that multifocal ring enhancement and diffusion restriction were the most common brain MRI changes. Diffusion restriction was mostly seen at the core of the lesion. Perivascular microbleeding seen on SWI and/or gradient-echo/T2* images, as well as infarction, were associated with later poor prognosis, whereas the distribution of the lesions was not predictive for outcome. 

We suggest including SWI sequences in routine diagnostic evaluation of immunocompromised children with suspected CNS-IMD.

## Figures and Tables

**Table 1:** Patient demographics and outcome.

Patients	Number
Female/Male	5/14
Age	Number
2- < 5 yr.	5
5- < 10 yr.	3
10–18 yr.	11
Outcome	Number
No severe neurological sequelae	7 (1 infarction)
Severe neurological sequela	5 (3 infarctions)
Deceased	7 (3 infarctions)
Pathogen	Number
*Aspergillus fumigatus*	9
*Aspergillus fumigatus, Rhizopus arrhizus*	1
*Rhizomucor pusillus*	1
Hyphae	5
Criteria of probable CNS-IMD without pathogen specified	3

**Table 2:** DWI and ADC findings and outcome:.

Outcome	Infarction	Central Restricted Diffusion	Rim Restricted Diffusion	Others
Total number of patients	7	10	3	2
Died	3	4	1	
Severe neurological sequelae	3	3	0	1 (cerebritis)
Favorable outcome	1 (focal pericallosal vasculitis)	3	2	1 (bleeding)

**Table 3:** T2*, SWI findings and outcome.

Outcome	Parenchymal Bleeding	Cluster of Microbleeds	Perivascular Microbleeding	Rim-Like	Vascular Blooming
Total number of patients	5	4	3	9	2
Died	2	1	2	4	1
Severe neurological sequelae	0	2	1	2	0
Favorable outcome	3	1	0	3	1

**Table 4:** Focal and multifocal involvement, enhancement pattern, and outcome.

Outcome	Parenchymal Multifocal (Lobes)	Parenchymal 1 lobe	Enhancement Ring-Like	Enhancement Nodular	Enhancement Subependymal No Ventriculitis	Enhancement Diffuse Parenchymal
Total number of patients	15	2	14	3	2	1
Died	7	0	5	1	1	0
Severe Neurological sequelae	2	1	3	1	1	1
Favorable outcome	6	1	6	1	0	0
